# The surgical safety checklist: a quantitative study on attitudes and barriers among gynecological surgery teams

**DOI:** 10.1186/s12913-021-07130-8

**Published:** 2021-10-16

**Authors:** Junming Gong, Yushan Ma, Yunfei An, Qi Yuan, Yun Li, Juan Hu

**Affiliations:** 1 Operating Room, West China Second University Hospital, Sichuan University/West China School of Nursing, Sichuan University; Key Laboratory of Birth Defects and Related Diseases of Women and Children (Sichuan University), Ministry of Education, Chengdu, Sichuan P. R. China; 2 Department of Anesthesiology, West China Second University Hospital/West China School of Medicine, Sichuan University; Key Laboratory of Birth Defects and Related Diseases of Women and Children (Sichuan University), Ministry of Education, Chengdu, Sichuan P. R. China; 3 Department of Laboratory Medicine, West China Hospital of Sichuan University, Chengdu, Sichuan P. R. China; 4 West China School of Nursing, Sichuan University, Chengdu, Sichuan P. R. China

**Keywords:** Surgical safety checklist, Patient safety, Operating room, Gynecologists, Anesthesiologists, Operating room registered nurses

## Abstract

**Background:**

Implementation of the surgical safety checklist (SSC) plays a significant role in improving surgical patient safety, but levels of compliance to a SSC implementation by surgical team members vary significantly. We aimed to investigate the factors affecting satisfaction levels of gynecologists, anesthesiologists, and operating room registered nurses (OR-RNs) with SSC implementation.

**Methods:**

We conducted a survey based on 267 questionnaires completed by 85 gynecologists from 14 gynecological surgery teams, 86 anesthesiologists, and 96 OR-RNs at a hospital in China from March 3 to March 16, 2020. The self-reported questionnaire was used to collect respondent’s demographic information, levels of satisfaction with overall implementation of the SSC and its implementation in each of the three phases of a surgery, namely sign-in, time-out, and sign-out, and reasons for not giving a satisfaction score of 10 to its implementation in all phases.

**Results:**

The subjective ratings regarding the overall implementation of the SSC between the surgical team members were different significantly. “Too many operations to check” was the primary factor causing gynecologists and anesthesiologists not to assign a score of 10 to sign-in implementation. The OR-RNs gave the lowest score to time-out implementation and 82 (85.42%) did not assign a score of 10 to it. “Surgeon is eager to start for surgery” was recognized as a major factor ranking first by OR-RNs and ranking second by anesthesiologists, and 57 (69.51%) OR-RNs chose “Too many operations to check” as the reason for not giving a score of 10 to time-out implementation. “No one initiates” and “Surgeon is not present for ‘sign out’” were commonly cited as the reasons for not assigning a score of 10 to sign-out implementation.

**Conclusion:**

Factors affecting satisfaction with SSC implementation were various. These factors might be essentially related to heavy workloads and lack of ability about SSC implementation. It is advisable to reduce surgical team members’ excessive workloads and enhance their understanding of importance of SSC implementation, thereby improving surgical team members’ satisfaction with SSC implementation and facilitating compliance of SSC completion.

## Background

In 2008, the World Health Organization (WHO) officially introduced the surgical safety checklist (SSC) to operating room health care providers [1]. As part of the “Safe Surgery Saves Lives” campaign initiated by the WHO, the SSC released in 2009 aimed to raise medical staff’s awareness of safety management in surgery. After a decade of development, the SSC has become an important tool for health care providers to ensure surgical patient safety in hospitals worldwide [2]. No doubt implementing the SSC has played a significant role in improving surgical patient safety [3]. Furthermore, there is sufficient evidence showing that the patients who require surgery are indicated to be under high-risk adverse events. De Vries, et al. [4] concluded that 10% of patients experienced medically related adverse events, and of which 40% were related to surgery. The international multi-center prosepective study by Haynes, et al. [5] has showed that implementation of the SSC reduced the incidence of surgical complications by 4% and the total hospital mortality rate by 0.7%, and the incidence of surgical site infections and accidental re-operation rate were also significantly reduced. There are sufficient high-level evidence-based data to support implementing the SSC in clinical practice [6]. However, after more than ten years of clinical application of the SSC, how to implement the SSC properly is still a major challenge [7–12]. Moreover, a good surgical safety is highly dependent on the rigorous, correct, and complete implementation of the SSC that could better reflect its clinical value [13]. In reality, the process of implementing the SSC, complied by surgeons, anesthesiologists, and nurses varies significantly, and pass rates of filling out the SSC also differ [14]. A meta-analysis found that the overall compliance rate of SSC implementation was between 12 and 100% (mean: 75%), and the compliance rate of SSC time-out was between 70 and 100% (mean: 91%) [15]. Besides, SSC completion needs to be improved. Tian, et al .[16] stated that even these safety checks were performed, 24% of the checklist items may still be missed. Aveling, et al. [17] observed that although medical staff could realize or appreciate the value of this checklist, they also felt puzzled or dissatisfied with its implementation, which related to conflicts between their philosophical background, local culture, medical education conditions, and social/economic factors. There are large differences in medical staff’s compliance to implement this worldwide checklist based on different medical conditions and cultures in different countries and regions. This situation may be observed in health care facilities more easily in China because there are significant regional differences. China has a vast land area and the largest popuation in the world. Since the SSC was introduced globally by WHO in 2008, the status of the checklist implementation has been not optimistic, which is a challenge faced by global surgical teams [2, 6–29]. In 2009, WHO introduced the SSC to China. On March 17, 2010, the Ministry of Health of China officially issued a document requiring the promotion and implementation of the SSC throughout the nation. In 2011, the SSC was included in the “Standard of Three-graded General Hospital Evaluation (2011 Edition)” in our health care system. In November 2016, the Legal Affairs Department of the National Health Commission promulgated the “Medical Quality Management Act”, and the SSC was regarded as one of the eighteen core systems to maintain medical quality and safety. So far, it has been applied and enforced as a core system for decade but the reality of the SCC implementation was not certain and clear. Therefore, we investigated satisfaction levels of gynecologists, anesthesiologists, and operating room registered nurses (OR-RNs) with SSC implementation to find key conflicting factors associated with SSC implementation between different surgical team members.

## Methods

### Organizational context

The West China Second University Hospital, Sichuan University, directly supervised by the National Health Commission of China, as the National Children’s Medical Centre in Western China is not only a specialized hospital for women and children but also a teaching hospital offering clinical teaching sites and internship opportunities for medical students from the West China School of Medicine, Sichuan University. It is the best obstetrics and gynecology continuing medical education base in Western China. The hospital undertakes a heavy workload in clinical affairs, such as treatment, referral, and consultation of critically ill patients, and has established the first medical alliance network of gynecology, obstetrics, and pediatrics in Western China. It undertakes social responsibilities in medical education, clinical training, scientific research, medical quality supervision, and quality management in the medical alliance. Our pilot study was organized by the Medical Administration Office of the West China Second University Hospital and the head nurse team from operating room. We distributed this survey though “WeChat” which is the most popular social media in China. All the staff who daily performed or assisted at surgeries in our operating room were the potential participants.

### The use of the surgical safety checklist in the West China Second University Hospital

The surgical safety checklist exists digitally in our hospital’s medical information system. Whenever surgeon submits a surgical operation application, the system automatically generates the patient’s surgical safety checklist based on medical information and surgical procedure. The surgeon prints the checklist and takes it with the patient to the operating room on the day of surgery. During the surgery, the three phases of a surgery, namely sign-in, time-out, and sign-out, are initiated successively based on the rules established by the Medical Administration Office of our hospital.

### Survey setup

After receiving messages that surgical pathology sample mislabeling occasionally occurred and patients were sent to the wrong wards after surgeries, the OR-RNs management team found that the SSC implementation was objectively incomplete during daily sugical procedures. The team members observed that some steps and content were even missing. Therefore, the operating room safety management team members worked on this issue based on those phenomena and conducted a review of past ten years’ relevant literature. Under the plan for the period from April 2020 to March 2022, the operating room safety management team worked jointly with the Medical Administration Office, the Department of Anesthesiology, and the Department of Gynecology to build a team carrying out a continuous quality improvement project, namely “improve the surgical safety checklist implementation based on multidisciplinary cooperation”. This survey was the fundamental of this project and will provide guidance for further investigation and intervention.

### Questionnaire setup

This single-page questionnaire was composed of 13 questions in total, in which 6 questions regarding respondent’s gender, age, length of service in medical practice, job position, educational background, and job title. 4 questions regarding respondent’s level of overall satisfaction with implementation of the SSC and its implementation in each of the three phases of surgery: sign-in, time-out, and sign-out. 3 questions regarding the reasons for not assigning a satisfaction score of 10 (if applicable) to SSC implementation in three phases of surgery. Respondent was required to use a rating scale of 0–10 to score levels of satisfaction, where 0 was the lowest level and 10 meant perfect. Close-ended questions were used to gather information about the reasons for not assigning a score of 10 to sign-in and time-out. All the close-ended questions’ response options were from the surgical safety management team of operating room (including gynecologists, anesthesiologists and OR-RNs), and all the response options were based on routine inspection data. The head nurse team summarized and analyzed those data, and found out the common factors influencing “sigh-in” and “time-out” implementation (these common factors might come from dissatisfaction among surgical team members). After surgical safety management team members discussed those response options, the questions and response options including the reasons for not assigning a score of 10 were selected and set up for the questionnaire. An open-ended question that was used to gather information about the reasons for not assigning a score of 10 to sign-out had a total of 5 response options including “other reasons”. The respondent who selected “other reasons” was optional to enter the detailed reason.

### Survey distribution

We shared a quick response code for the self-reported questionnaire to WeChat groups of the gynecologists, anesthesiologists, and OR-RNs. The quick response code and questionnaire were produced using an online survey application called “WENJUANXING”. Respondents could access the questionnaire by scanning the quick response code. No incentives were provided for respondents to complete the questionnaire.

All methods were carried out in accordance with relevant guidelines and regulations. Ethical approval of this study was obtained from the Medical Ethics Committee of the West China Second University Hospital, Sichuan University [YXKY2020LSP(140)]. The purpose of the study was stated at the beginning of the questionnaire. Respondents were not required to enter their names for the questionnaire. The questionnaires were voluntarily and anonymously filled out by respondents. The respondents who completed and submitted the questionnaires were regarded as verbally agreeing to participate in the study. The procedure of obtaining verbal consent was approved by the Medical Ethics Committee of the West China Second University Hospital. All data collected were kept strictly confidential and not be used for any purpose other than this study.

### Date collection

Our survey was piloted and covered all the anesthesiologists and OR-RNs working in our facility, and team of surgeons which consisted of employees, obstetrics and gynecology residents, and continuing medical education trainees. A total of 267 questionnaires completed by 85 gynecologists from 14 gynecological surgery teams, 86 anesthesiologists, and 96 OR-RNs were collected. Incomplete questionnaires could not be submitted successfully and were not accessible to the researchers. The average time to complete the questionnaire was 238.85 s (standard deviation (SD) = 245 s; inter-quartile range (IQR) = 134 s; range: 59–2193 s), and the median time was 176 s.

### Statistical methods

SPSS statistical software 21.0 (IBM Corporation, Armonk, NY, USA) was used for data analysis. Measurement data, conforming to a normal distribution, were expressed as the mean ± standard deviation; otherwise, the median and inter-quartile range were used. Enumeration data were described in percentage composition. The enumeration data were analyzed with the Kruskal-Wallis test and *P* < 0.05 was used to define a statistically significant difference in surgical team members’ satisfaction levels with SSC implementation. .

## Results

Demographic information of the gynecologists, anesthesiologists, and OR-RNs who participated in the study is shown in Table 1.

**Table 1:** Demographic information of gynecologists, anesthesiologists and operating room registered nurses

	Gynecologists (*n* = 85)	Anesthesiologists (*n* = 86)	OR-RNs (*n* = 96)
Gender (female, %)	60 (70.59%)	68 (79.07%)	90 (93.75%)
Age (median, range, IQR)	33 (22–54, 9)	31 (21–54, 9)	29 (23–59, 8)
Length of service in medical practice (n, %)			
0–5 years	36 (42.35%)	45 (52.33%)	46 (47.92%)
6–10 years	20 (23.53%)	17 (19.77%)	21 (21.88%)
11–20 years	20 (23.53%)	16 (18.60%)	22 (22.92%)
> 20 years	9 (10.59%)	8 (9.3%)	7 (7.28%)
Educational background (n, %)			
Associate Degree	/	/	9 (9.38%)
Bachelor’s degree	9 (10.59%)	33 (38.37%)	83 (86.46%)
Master’s degree	30 (35.29%)	38 (44.19%)	4 (4.16%)
MD/PhD	46 (54.12%)	15 (17.44%)	/
Job title (n, %)			
Resident/RN	29 (34.12%)	49 (56.98%)	67 (69.79%)
Attending/advanced RN	36 (42.35%)	21 (24.42%)	28 (29.17%)
Associate professor and above	20 (23.53%)	16 (18.6%)	1 (1.04%)

### Overall satisfaction with implementation of the surgical safety checklist

The mean scores of SSC implementation satisfaction in gynecologists, anesthesiologists, and OR-RNs were 9.14, 8.77, and 8.36, respectively. There was a statistically significant difference in overall satisfaction with SSC implementation between the three groups (*P* = 0.001) (Table 2).

**Table 2:** Satisfaction with implementation of the surgical safety checklist

	Gynecologists (*n =* 85) mean (median, range, IQR)	Anesthesiologists (*n =* 86) mean (median, range, IQR)	OR-RNs (*n =* 96) mean (median, range, IQR)	*P* value
Satisfaction with overall implementation of surgical safety checklist	9.14 (10, 3–10, 1)	8.77 (9, 5–10, 2)	8.36 (9, 3–10, 2)	0.001
Satisfaction with sign-in implementation	9.66 (10, 5–10, 2)	9.49 (10, 5–10, 1)	9.6 (10, 3–10, 0)	0.097
Satisfaction with time-out implementation	8.07 (9, 0–10, 3)	7.6 (8, 0–10, 4)	6.72 (7, 1–10, 4)	0.000
Satisfaction with sign-out implementation	7.6 (9, 0–10, 5)	7.12 (8, 0–10, 5)	7.8 (8, 0–10, 4)	0.238

### Satisfaction with implementation of the surgical safety checklist in sign-in phase

The mean scores of “sign-in” implementation satisfaction in gynecologists, anesthesiologists, and OR-RNs were 9.66, 9.49, and 9.6, respectively. There were no significant differences in satisfaction with sign-in of SSC implementation between the three groups (*P* = 0.097) (Table 2).

Among the 267 respondents, 54 (20.22%) of them, consisting of 11 (12.9%) gynecologists, 23 (26.74%) anesthesiologists, and 20 (20.83%) OR-RNs, did not assign a full score. “Too many operations to check” ranked first among the reasons for gynecologists and anesthesiologists not to assign a full score to “sign-in” implementation, and “Surgeon is not present for ‘sign in’” ranked first among the reasons for OR-RNs not to assign a full score (Table 3).

**Table 3:** Reasons for not assigning a score of 10 to sign-in, time-out, and sign-out

	Gynecologists (*n =* 85)	Anesthesiologists (*n =* 86)	OR-RNs (*n =* 96)
**Reasons for not assigning a score of 10 to sign-in implementation (n, %)**	11 (12.9%)	23 (26.74%)	20 (20.83%)
Too many operations to check	4 (36.36%)	16 (60.87%)	10 (50%)
Anesthesiologist is not present for “sign in”	3 (27.27%)	1 (4.35%)	4 (20%)
Surgeon is not present for “sign in”	3 (27.27%)	13 (56.52%)	16 (80%)
OR-RNs is not present for “sign in”	1 (9,09%)	2 (8.7%)	1 (5%)
No one initiates	2 (18.18%)	3 (13.04%)	14 (70%)
**Reasons for not assigning a score of 10 to time-out implementation n, %)**	46 (54.12%)	60 (69.77%)	82 (85.42%)
Too many operations to check	20 (43.48%)	24 (40%)	57 (69.51%)
Surgeon is eager to start for surgery	1 (2.17%)	26 (43.33%)	66 (80.49%)
Hard to ensure the three groups implement the checklist properly	20 (43.48%)	40 (66.67%)	48 (58.54%)
Too many checklist items interfere with schedule of surgical operations	18 (39.13%)	24 (40%)	25 (30.49%)
No one initiates	/	17 (28.33%)	50 (60.98%)
Do not know what “time out” is	1 (2.17%)	/	/
The operation is extremely urgent, and there is no time to check	/	/	2 (2.44%)
**Reasons for not assigning a score of 10 to sign-out implementation (n, %)**	45 (52.94%)	58 (67.44%)	58 (60.42%)
No one initiates	27 (60%)	31 (53.45%)	34 (58.62%)
Anesthesiologist is not present for “sign out”	3 (6.67%)	/	4 (6.9%)
Surgeon is not present for “sign out”	16 (35.56%)	39 (67.24%)	50 (86.21%)
OR-RNs is not present for “sign out”	2 (4.44%)	/	1 (1.72%)
Other reasons	10 (22.2%)	6 (10.34%)	1 (1,72%)

### Satisfaction with implementation of the surgical safety checklist in time-out phase

The mean scores of time-out implementation satisfaction in gynecologists, anesthesiologists, and OR-RNs were 8.07, 7.6, and 6.72, respectively. We observed a large discrepancy between the three groups (*P* = 0.000) (Table 2).

Among the 267 respondents, 188 (70.41%) of them, consisting of 46 (54.12%) gynecologists, 60 (69.77%) anesthesiologists, and 82 (85.42%) OR-RNs, did not assign a full score. “Too many operations to check” and “Hard to ensure the three groups implement the checklist properly” ranked first among the reasons for gynecologists not to assign a full score; “Hard to ensure the three groups implement the checklist properly” and “Surgeon” is eager to start for surgery” ranked first among the reason for anesthesiologists and OR-RNs, respectively (Table 3).

### Satisfaction with implementation of the surgical safety checklist in sign-out phase

The mean scores of sign-out implementation satisfaction in gynecologists, anesthesiologists, and OR-RNs were 7.60, 7.12, and 7.8, respectively. There were no statistically significant differences in the sign-out satisfaction scores between the three groups (*P* = 0.238). The three groups’ mean satisfaction score of sign-out implementation was the lowest among those of SSC implementation in the three phases (Table 2).

Among the 267 respondents, 161 (60.30%) of them, consisting of 45 (52.94%) gynecologists, 58 (67.44%) anesthesiologists, and 58 (60.42%) OR-RNs, did not assign a full score. Factors negatively influencing the gynecologists’ satisfaction most was “No one initiates”, and followed by “Surgeon is not present for ‘sign out’”; 10 (22.2%) surgeons chose “Other reasons” and 7 of them provided the details; 1 surgeon (MD/Ph.D, attending, length of service: 0–5 years) entered “It may not be necessary to sign out”; 1 (MD/Ph.D, associate professor, length of service: 11–20 years) entered “It is not necessary to sign out”; and, 1 (MD/Ph.D, professor, length of service: > 20 years) entered “Sign out is completely unnecessary”. These three reasons manually entered by respondents could be summarized as “Sign out is unnecessary”. Two gynecologists who were attending physicians and held master’s degrees and 6–10 years’ medical work experience specified “Because postoperative surgical pathology specimens need to handle and a large amount of paperwork need to fill out, I could not be present for‘sign out’”; 1 specified “I do not know when to carry out the sign-out”, and 1 gynecologist (MD/Ph.D) with a senior title and more than 20 years’ medical work experience specified “I leave the operating room immediately after the surgery and I do not know that ‘sign out’ is needed”.

Among the 58 anesthesiologists who did not assign a full score to “sign-out” implementation, “Surgeon is not present for ‘sign out’” and “No one initiates” were the two major factors; 6 (10.34%) anesthesiologists chose“Other reasons”. Of them, 1 specified “Have no awareness of sign-out phase”, and 1 (MD/Ph.D, associate professor, length of service > 20 years) entered “Sign-out is not related to anesthesia”.

The major factors negatively influencing the OR-RNs’ satisfaction was “Surgeon is not present for ‘sign out’” and “No one initiates” also was a core issue. The OR-RNs who selected “Other reasons” did not specify the reason.

## Discussion

Our study has shown that China’s SSC implementation status is not optimistic, and the mutual recognition between different teams is controversial. The difference in SSC subjective satisfaction and the differentiated attitudes about influencing factors indicate that there were significant barriers to communication between those team members. Zhu, et al. [19] reported in their study that the implementation rates of “sign in”, “time-out”, and “sign out” were 93.35, 78.22, 64.26%, respectively after 10 years of implementation of the SSC in China, and those data were obtained from the 2019 annual meeting of the Chinese Association of Anesthesiologists. This was an evaluation from the perspective of anesthesiologists, and the result coincides with our survey. Both of them indicated that satisfaction with the SCC implementation in the three phases gradually declined with the process of the surgery. It can be inferred that although our survey was conducted in a single teaching hospital in southwest China, it represented the actual situation in China and the influencing factors are various.

Sexton, et al. [30] introduced a validated tool called safety attitude questionnaire (SAQ) which was used to measure attitudes and perceptions in various safety-related issues in healthcare. Our survey method was inspired by this tool and we cheered up surgery participants to rating how satisfied they were about SSC implementation. In our survey, satisfaction with overall implementation of the SSC was quite different between gynecologists, anesthesiologists, and OR-RNs, which revealed that significant differences might exist in the mutual recognition, attitude, and their perception. It was a bad indicator that deduced a possible antagonistic relationship between surgical team members. Research by Haynes, et al. [2, 13, 20] has shown that a healthy relationship between the surgical teams is essential to a complete and smooth implementation of the SSC, and has been proven in many other studies.

In the “sign in” phase, the mean scores of the satisfaction between the three groups were high and no statistically significant differences were found, which demonstrated a good status of the “sign in” initiated by the anesthesiologist. However, 54 respondents did not give a full score. The most selected factors were “Too many operations to check” and “Surgeon is not present for ‘sign in’”. Looking back to our hospital’s workload statistics in 2019, the average workload of each operating room was 5.5 patients per day, 1-8 h for each patient, and occupied 10.5–12.5 h per day. A high surgery demands in our area caused a huge workload to surgeons, and we observed this attitude “Too many operations to check”. The subjective emotions and stress state of the participants especially the surgeons and anesthesiologists significantly affect the implementation of the “sign in” phase. Gillespie, et al. [21] observed the same phenomenon in their study and got a similar conclusion. We recognized that surgery is a time-sensitive and complex environment [2]. The reality is that most of gynecologists who treat patients with malignant tumors such as teratomas, cervical cancer, ovarian cancer, or uterine fibroids in our hospital are responsible for improving the quality of life even saving lives. So, they might be under tremendous pressure during the day of the surgery both from their responsibilities to the patients and the workload. They are reluctant to fill in the documents or unnecessary operation checks because they think such procedure is too trivial and may interfere with the progress of the surgery. They are always in a hurry to fulfill their own tasks on the day of surgery. This phenomenon was also observed by OR-RNs as “Surgeon is not present for ‘sign in’”. Our study reflected that the surgeons, anesthesiologists, and OR-RNs were under different levels of work stress during sign-in phase and implementation of the “sign in” was substantially affected under stress. These differences in subjective cognition might arise from different job roles and duties, but all the outcomes or benefits from the surgery was based on the safety. Singh, et al. [6] suggested that surgeons should be familiar with, advocate for the use of, and participate in all three phases of the surgical safety checklist in order to improve patient safety. Balancing clinical workload as much as possible and strengthening surgical team members’ participation in “sign in” may improve their compliance with the sign-in completion. Research by Haynes, et al. [2] has shown that barriers to SSC implementation can be overcome with education, practice, and leadership.

Core principle of the time-out is “perform at the correct site in the correct patient through the correct procedure all times” [22, 23]. In our study, we found that the difference in satisfaction with time-out implementation between gynecologists, anesthesiologists, and OR-RNs was most significant, and 70.41% of respondents did not assign a full score. “Too many operations to check” was identified as the top-rated factor for the gynecologists, but not for anesthesiologists and OR-RNs. The top-rated factor for OR-RNs and anesthesiologists was “Surgeon is eager to start for surgery”. Surgeon is the initiator in time-out phase. Low level of satisfaction with time-out implementation in this phase must be related to surgeon’s weak awareness of “time out”, and so it is not difficult to understand why we were facing this situation. WHO has indicated that in order to successfully implement the SSC, the chiefs of surgery, anesthesia and nursing departments must publicly embrace the belief that patient safety is a priority and that use of the SSC can help improve patient safety. If there is no demonstrable leadership, instituting such a checklist may breed discontent and antagonism [18]. Unfortunately, we found such signs in our investigation. More importantly, the surgical team must carefully consider which staff member is most suitable for this role. There is a circulating nurse in many surgical teams, but any healthcare professional could coordinate the checklist process [24]. Dillon [22] pointed out that circulating nurse should be the most suitable candidate.

In our survey, a gynecologist, who had worked for 0–5 years and hold a master’s degree, did not know that “time out” was a necessary procedure. It is speculated that this gynecologist might not receive effective training about the SSC or not fully participate in the “time out” process. Because SSC training was organized by each team independently in our hospital, it is necessary to investigate the quality of training and assess training effectiveness. As many of the gynecologists, anesthesiologists, and OR-RNs who did not assign a score of 10 believed that “Too many checklist items interfere with the schedule of surgical operations” was another important factor, it is advisable to revise the checklist to meet the need for gynecological surgery especially. The WHO implementation manual indicates that each locale is encouraged to reformat, reorder or revise the checklist to accommodate local practice while ensuring completion of the critical safety steps in an efficient manner [18]. So we will need to improve and optimize the SSC according to the characteristics of gynecological surgery.

More than half of the gynecologists, anesthesiologists, and OR-RNs did not satisfy with the “sign out” with the lowest mean score. The “No one initiates” was a core factor and “Surgeons is not present for ‘sign out’” ranked second. “Sign out” should be initiated by OR-RNs but it failed in reality. 17 respondents selected “Other reasons”, and 8 of whom gave descriptions. Three thought that “sign out” was unnecessary; two thought that they had to deal with specimens and a large amount of paperwork after surgery and could not be present for “sign out”, one did not know when to initiate “sign out”, and one usually immediately left the operation room after surgery. To our surprise, a gynecologist who had a doctorate, a senior professional title, and more than 20 years’ work experience did not know what “sign out” was. Survey results indicated that the lack of surgeon’s participation in this phase might be the most important factor influencing OR-RNs to initiate this step. Those phenomena also indicated that those surgeons might lack of education about the meaning of the SSC. The WHO SSC manual tells us that the safety steps should inspire surgery teams to comply with each element of this checklist. Besides, each team member should respect this procedure [18].

The WHO recommends that the SSC should be completed jointly by surgeons, anesthesiologists, and OR-RNs. There is ample evidence indicated that unobstructed and effective communication between surgical team members can enhance the safety of surgery [6]. Lingard, et al. [25] pointed out that poor communication between surgical team members directly affects patient’s safety. Most important factors were related to surgeons in our study. The Canadian Association of Obstetricians and Gynecologists recommends that surgeons should understand and be familiar with all the three parts of the SSC and should fully support its implementation in clinical practice [6]. Implementing the SSC without connotation or just for completion is meaningless and worthless. The ultimate value of the SSC implementation should be delivering a safer environment for our surgical patients and also protecting our medical team.

The value of SSC implementation is still evolving amidst controversy. Sendlhofer, et al. [31] pointed out that constant dialectical thinking and evaluation of this checklist was the driving force for its continuous improvement. Moreover, the core values of patient safety are continuously strengthened and rooted in health care providers [8, 10, 26–29, 32]. Furthermore, continuous quality improvement will always accompany with our clinical operations and procedures. By having a keen perception in clinic and continuously pursuing the surgical safety and quality improvement, we will create a safer surgical environment for both our patients and surgical teams.

## Limitations

Factors influencing SSC implementation include but are not limited to the ones identified in our study. Studies of other factors and applying interventions to improve surgical team members’ awareness of the SSC implementation are needed. Our investigation and research involved very superficial and subjective scoring and evaluation. We did not conduct an in-depth analysis and research on the underlying factors. Therefore, there is no improvement and evaluation for the problem, which is the limitation in our study. The actual application status of our SSC at the present stage is not optimistic, and surgical team members may not pay attention to corresponding improvements because no serious patient safety risks have occurred. The results from our investigation provide specific goals and directions for our further research and continuous quality improvement.

## Conclusion

Factors affecting surgical team members’ satisfaction with SSC implementation were various. These factors might be essentially related to heavy surgical workloads and lack of ability about SSC implementation. It is advisable to reduce workload of the surgical team members who have excessive workloads, enhance surgical team members’ understanding of the importance of SSC implementation and the meaning of each step of this checklist, and provide them intensive training on compliance of SSC completion, thereby improving surgical team members’ satisfaction with SSC implementation and facilitating compliance of SSC completion.

## Data Availability

The datasets used and/or analysed during the current study are available from the corresponding author on reasonable request.

## Abbreviations

OR-RNs: Operating room registered nurses
SSC: Surgical safety checklist
WHO: World Health Organization
